# Longevity of antibody and T-cell responses against outer membrane antigens of *Orientia tsutsugamushi* in scrub typhus patients

**DOI:** 10.1038/emi.2017.106

**Published:** 2017-12-20

**Authors:** Na-Young Ha, Yuri Kim, Chan-Ki Min, Hong-Il Kim, Nguyen Thi Hai Yen, Myung-Sik Choi, Jae-Seung Kang, Yeon-Sook Kim, Nam-Hyuk Cho

**Affiliations:** 1 Department of Microbiology and Immunology, Seoul National University College of Medicine, Seoul 03080, Republic of Korea; 2 Department of Biomedical Sciences, Seoul National University College of Medicine, Seoul 03080, Republic of Korea; 3 Department of Microbiology, Inha University School of Medicine, Incheon 22212, Republic of Korea; 4 Division of Infectious Diseases, Department of Internal Medicine, Chungnam National University School of Medicine, Daejeon 35015, Republic of Korea; 5 Institute of Endemic Disease, Seoul National University Medical Research Center and Bundang Hospital, Seoul 03080, Republic of Korea

**Keywords:** antibody, *Orientia tsutsugamushi*, scrub typhus, T cell, vaccine

## Abstract

Scrub typhus, caused by *Orientia tsutsugamushi* infection, has been a serious public health issue in the Asia-Pacific region, with rising incidence and sporadic outbreaks. However, human protective immunity against specific antigens has been poorly characterized for this bacterium. In addition, immunity produced in early vaccine trials or even after natural infections, did not last long and had poor cross-reactivity among various genotypes. Here, we systematically investigated the kinetics and magnitude of specific adaptive immunity against two membrane antigens, 56 kDa type-specific antigen (TSA56) and surface cell antigen A (ScaA), that are involved in bacterial adhesion and invasion of the host in 64 recovered scrub typhus patients. Antibody responses to the bacterial antigens in patients were generally short-lived and waned to baseline levels 2 years after recovery. The anti-TSA56 IgG responses were predominantly composed of the IgG1 and IgG3 subclasses and persisted for up to 1 year after recovery, whereas IgG specific to ScaA primarily consisted of more transient IgG1, with limited responses by other subclasses. Cellular immunity, including CD4 and CD8 T-cells specific to membrane antigens, also rapidly declined from 1 year after infection, as measured by enzyme-linked immunospot (ELISPOT) assays and flow cytometry. The short longevity of antigen-specific adaptive immunity might be attributable to limited memory responses, as observed in earlier vaccine studies using whole bacterial antigens. Finally, we identified HLA-A*0201-restricted and highly conserved CD8 T-cell epitopes in the TSA56 antigen, which may be valuable tools for assessing cellular immunity against *O. tsutsugamushi* and developing an effective scrub typhus vaccine.

## INTRODUCTION

Scrub typhus is an acute febrile infectious disease endemic in Asia and the western Pacific region.^1^ The disease has been a serious public health problem, afflicting more than one million patients annually in the endemic area. In addition, a rapid increase in disease incidence, as well as sporadic local outbreaks, has been reported in several endemic countries within the last decade.^1^ Recently, scrub typhus has emerged in unexpected geographic locations, including Chile^2^ and Kenya,^3, 4^ suggesting that the endemicity of scrub typhus may not be restricted to the traditional ‘tsutsugamushi triangle’ in the Asia-Pacific area.^5^ Therefore, more attention needs to be paid to this neglected infectious disease.

Scrub typhus is a mite-borne infectious disease that is caused by *Orientia tsutsugamushi*, an obligate intracellular bacterium.^6^ Bacteria are transmitted from infected chigger mites to humans and systemically spread to multiple organs, including the lung, kidney, liver and spleen.^7^ Clinical presentations are typically characterized by eschar at the mite-biting site, fever, rashes, lymphadenopathy and myalgia. The disease severity can vary from a self-limiting flu-like syndrome to a life-threatening disease causing multi-organ failure and disseminated intravascular coagulation.^8^ The mortality of untreated patients is ~6%, with a wide range of 0–70% (min–max) depending on the endemic area and patient’s age.^9^ Even though early diagnosis and subsequent proper treatment with antibiotics, such as doxycycline or chloramphenicol, usually results in rapid recovery from febrile illness, reports of poorly responsive cases to antibiotic therapy suggest possible drug resistance.^10, 11^ In addition, recurrent human infection has often been observed in highly endemic regions,^12, 13^ primarily due to antigenic heterogeneity and the short longevity of adaptive immunity against the bacterial pathogen.^8^ Despite the wide range of preventative approaches that have been attempted in the past 70 years, all have failed to yield an effective prophylactic vaccine.^8^

In earlier vaccine studies using inactivated bacterial antigens or live *Orientia* challenge followed by antibiotic treatment, protective immunity against the homologous strain lasted for only a few years, whereas heterologous protection generally disappeared within a few months in humans.^14^ Although there is very little information about protective immunity in humans, antibodies generated from mice immunized with outer membrane proteins of *O. tsutsugamushi*, such as 56 kDa type-specific antigen (TSA56) and surface cell antigen A (ScaA), neutralized and significantly suppressed bacterial entry into host cells *in vitro*.^15, 16^ In addition, we recently found that immunization with ScaA, when combined with TSA56, not only provides protective immunity against lethal challenges with the homologous strain but also confers significant protection against heterologous strains in a mouse infection model.^16^ Recently, two other groups reported that CD8 T cells from infected mice protected naive mice from lethal challenges with *O. tsutsugamushi*.^17, 18^ These results indicate that antigen-specific adaptive immunity, especially bacteria-specific CD8 T cells, may have a significant role in protecting against scrub typhus. Nevertheless, antigen-specific adaptive immune responses and their longevity are poorly characterized in scrub typhus patients. For example, immunoglobulin G (IgG) is classified into four subclasses (IgG1 ~4) according to their constant regions and variable degree of effector functions, such as complement fixation, neutralization and opsonization.^19^ However, a systemic study of IgG subtypes specific to *O. tsutsugamushi* antigens has not been conducted in human patients. Although a few studies have shown that IgG responses in recovered scrub typhus patients only persist for a short period,^12, 20, 21^ the majority of these studies used whole bacteria as antigens for immunofluorescence assays (IFA), and antibody responses against specific protein antigens were measured by the total IgG or IgM level.^22^ Moreover, CD4 or CD8 T-cell responses specific to protein antigens of *O. tsutsugamushi* have been poorly characterized in scrub typhus patients, despite their functional importance in protective immunity. In this report, we systematically investigated specific adaptive immunity against two *Orientia* membrane antigens, TSA56 and ScaA, in recovered patients to assess the dominant subtypes of the IgG and T-cell responses as well as their kinetic changes after recovery. We also screened and selected potential CD8 T-cell epitopes of TSA56 as they can be applied as a useful tool for monitoring antigen-specific CD8 T-cell immunity in humans. The types, magnitudes and kinetics of specific adaptive immunity in scrub typhus patients may provide valuable clues for assessing functional adaptive immunity in humans and for the development of an effective vaccine.

## MATERIALS AND METHODS

### Ethics statement

Ethical approval for this work was granted by the Institutional Review Board of both Seoul National University Hospital (IRB NO 1308-058-513) and Chungnam National University Hospital (IRB NO 2014-12-006). All patients and healthy volunteers provided written informed consent prior to sample collection.

### Study subjects

Human peripheral blood was drawn from healthy volunteers (*n*=29) and recovered scrub typhus patients (*n*=64) after obtaining informed consent at Chungnam National University Hospital in Daejeon, South Korea. To analyze the humoral and cellular immune responses, scrub typhus patients were divided into four groups based on the time after recovery from the disease (Y0: within 2 weeks after recovery, Y1–Y3: 1–3 year(s) after recovery). Scrub typhus was confirmed based on clinical symptoms and a positive serology: a fourfold or greater rise in the titer of paired plasma or single cut-off titer of an IgM antibody ≥1:160 by an indirect immunofluorescence antibody assay (IFA) against *O. tsutsugamushi* antigens or passive hemagglutination assay (PHA) during hospital admission.^21^ Healthy volunteers had never been previously diagnosed with scrub typhus and their sera were negative when examined by IFA. The mean age of the scrub typhus patients was 60.1 years (range: 17–88) and healthy control was 60.5 years (range: 52–71). The ratio of males to females was 45:55 in scrub typhus patients and 32:68 in healthy controls.

### Preparation of peripheral blood mononuclear cells and plasma

Blood samples were collected in heparinized tubes and centrifuged at 800*g* for 20 min. The top layer containing plasma was cryopreserved to titrate antibodies against *O. tsutsugamushi* antigens. Peripheral blood mononuclear cells (PBMCs) were isolated by standard density centrifugation with Histopaque (GE Healthcare, Pittsburgh, PA, USA). Fresh PBMCs were used for Enzyme-Linked ImmunoSpot (ELISPOT) assays, and the remaining PBMCs were cryopreserved in liquid nitrogen for flow cytometric analysis.

### Preparation of recombinant ScaA and TSA56 proteins

The *scaA* and *tsa56* genes were amplified from genomic DNA of the *O*. *tsutsugamushi* Boryong strain (GenBank accession No. AM494475.1) by PCR using the primer pairs listed in Table 1. The PCR products were cloned into the pET-28a plasmid and sequenced to confirm successful in-frame cloning. Recombinant ScaA and TSA56 proteins were purified from *E*. *coli* BL21 (DE3) harboring a recombinant plasmid encoding each bacterial antigen. Following induction with isopropyl β-D-thiogalactoside (IPTG, 0.1 mM, Duchefa, Zwijndrecht, Netherlands) at 16 °C for 16 h, the proteins were purified using Ni-nitrilotriacetic acid (NTA) His-resin (Qiagen, Germantown, MD, USA) according to the manufacturer’s instructions. The purified proteins were dialyzed against phosphate-buffered saline (PBS) in Slide-A-Lyzer Dialysis Cassettes (Thermo Scientific, Waltham, MA, USA) at 4 °C overnight. The identity and purity of the proteins were assessed by western blotting and Coomassie blue staining as previously described.^23^

### CD8^+^ T-cell epitope prediction and HLA typing

To identify potential HLA-A*0201-restricted CD8 T-cell epitopes within the TSA56 antigen of *O. tsutsugamushi*, four web-based programs were used: BIMAS (https://www-bimas.cit.nih.gov/),^24^ SYFPEITHI (http://www.syfpeithi.de/),^25^ IEDB (http://tools.immuneepitope.org/) and NetMHCpan (www.cbs.dtu.dk/services/NetMHCpan/).^26^ We selected 17 potential peptide epitopes that were predicted with top 1% scores or percentile ranks in at least three of the four programs. The peptide sequences and their affinity (IC_50_) to HLA-A*0201 predicted by NetMHCpan are summarized in Table 2. Human leukocyte antigen (HLA) typing was performed to screen for the HLA-A2 type using a PE-labeled HLA-A2 antibody (MBL, Nagoya, Japan) by flow cytometric analysis.

### Immunofluorescence assay (IFA)

An IFA was performed to measure the total IgG antibody titers against *O. tsutsugamushi.* L929 cells infected with three strains of *O. tsutsugamushi* (Boryong, Karp and Gilliam strain) were harvested, mixed in equal amounts and used as antigens for IFA as previously described.^23^ Briefly, infected L929 cells were harvested, washed with PBS, spotted onto teflon-coated spot slides and fixed with cold acetone for 10 min. The slides were stored at -70 °C until use. Twofold serially diluted (1:40 to 1:5120 in PBS) patient plasma was added to the antigen-coated spot on the slide and incubated for 30 min in a moist chamber at room temperature. An Alexa Fluor 488-conjugated goat anti-human IgG antibody (Molecular Probes, Waltham, MA, USA), diluted 1:1000 in PBS, was used as the secondary antibody. The stained slides were examined under an Olympus FV1000 laser scanning confocal microscope (Olympus, Tokyo, Japan). The endpoint titer of IFA was defined as the highest titer showing a fluorescence signal above the background.

### ELISA

To assess the ScaA- or TSA56-specific antibody responses, 96-well immunoassay plates (Nunc, Waltham, MA, USA) were coated with 100 μL of purified antigen at a concentration of 1 μg/mL at 4 °C overnight. The plates were then blocked for 2 h at room temperature (RT) with PBS containing 5% skim milk. One hundred microliters of twofold serially diluted plasma samples were incubated for 2 h at RT. After washing with PBS containing 0.05% Tween20 (0.05% PBST), horseradish peroxidase (HRP)-conjugated mouse anti-human IgG1, IgG2, IgG3, IgG4 or IgG antibody (Southern Biotech, Birmingham, AL, USA) was added and incubated for 1 h at RT. Wells were washed with 0.05% PBST and incubated with a 3,3′,5,5′-tetramethylbenzidine (TMB) peroxidase substrate solution (KPL, Gaithersburg, MD, USA) for 10 min. The reactions were stopped by addition of a 1 M phosphoric acid solution. Absorbance was measured at 450 nm using a microplate reader (Beckman Coulter, Brea, CA, USA). The cut-off titer for the ELISA was determined as the lowest titer showing an optical density (OD) over the mean OD plus 3 × standard deviation (s.d.) from 19 control plasma samples (diluted 1:100).

### Flow cytometric analysis

Human PBMCs were cultured for 18–20 h in RPMI 1640 medium (Gibco, Gaithersburg, MD, USA) supplemented with 10% heat-inactivated fetal bovine serum (FBS), 50 nM β-mercaptoethanol, 100 U/mL penicillin/streptomycin and 2 mM glutamine (Welgene, Seoul, South Korea) in the presence of 10 μg/mL of purified ScaA or TSA56 antigens in 24-well, flat-bottomed culture plates (5 × 10^6^ cells per well). For intracellular cytokine staining, GolgiPlug (BD Biosciences, San Jose, CA, USA) was added and cells were further incubated for 6 h. Cells were then washed 3 times with ice-cold FACS buffer (PBS containing 1% bovine serum albumin and 1 mM EDTA) and blocked on ice for 30 min with ultra-block solution (10% rat sera, 10% hamster sera, 10% mouse sera and 10 μg/mL of 2.4G2 monoclonal antibody). PBMCs were stained with Pacific blue-conjugated CD4 and FITC-conjugated CD8 antibodies (BD Pharmingen, Franklin Lakes, NJ, USA) at 4 °C for 30 min. After surface CD4 or CD8 staining, cells were washed three times with ice-cold FACS buffer and subjected to intracellular cytokine staining using the Cytofix/Cytoperm kit according to the manufacturer’s instructions (BD Biosciences). Cells were stained with an APC-conjugated IFN-γ antibody (BD Pharmingen) at 4 °C for 30 min. The stained cells were analyzed with a FACS LSRII flow cytometer (BD Immunocytometry Systems, San Jose, CA, USA). Data were analyzed using FlowJo software (Tree Star, Ashland, OR, USA).

### Enzyme-linked immunospot (ELISPOT) assay

Human IFN-γ/IL-4 dual color ELISPOT assays were performed according to the manufacturer’s instructions (Abcam, Cambridge, UK). Briefly, polyvinylidene difluoride-backed microtiter plates (MSIP, Millipore, Temecula, CA, USA) were treated with 25 μl of 35% methanol for 30 s and washed 3 times with sterile PBS. Plates were coated with IFN-γ/IL-4 capture antibodies at 4 °C overnight. Human PBMCs (5 × 10^5^ cells per well) were stimulated with 10 μg/mL ScaA, TSA56 or 9-mer epitope peptides (1 μM) in the presence of co-stimulatory anti-CD28 and anti-CD49d monoclonal antibodies (BD Pharmingen) at 37 °C for 18 h. The wells were incubated further with a secondary anti-IFN-γ FITC-conjugated antibody and biotinylated anti-IL-4 antibody at room temperature for 90 min, followed by incubation with anti-FITC HRP and streptavidin-alkaline phosphatase (AP) conjugates for 1 h. Immunospots were developed by sequential incubation of wells with an AEC or BCIP/NBT substrate and were counted using a CTL ImmunoSpot reader (Cellular Technology, Cleveland, OH, USA). The count settings (intensity, gradient and size of spot) were manually confirmed by the naked eye in each experimental set.

### Statistical analysis

Data were analyzed using GraphPad Prism 5.01 software (GraphPad Software, La Jolla, CA, USA). Statistical analysis was performed using one-way analysis of variance (ANOVA), followed by the Newman–Keuls *t*-test for comparisons of values among different groups. *P*<0.05 was considered statistically significant.

## RESULTS

### Kinetics and magnitude of the anti-ScaA and TSA56 antibody responses in scrub typhus patients

**Figure 1:**
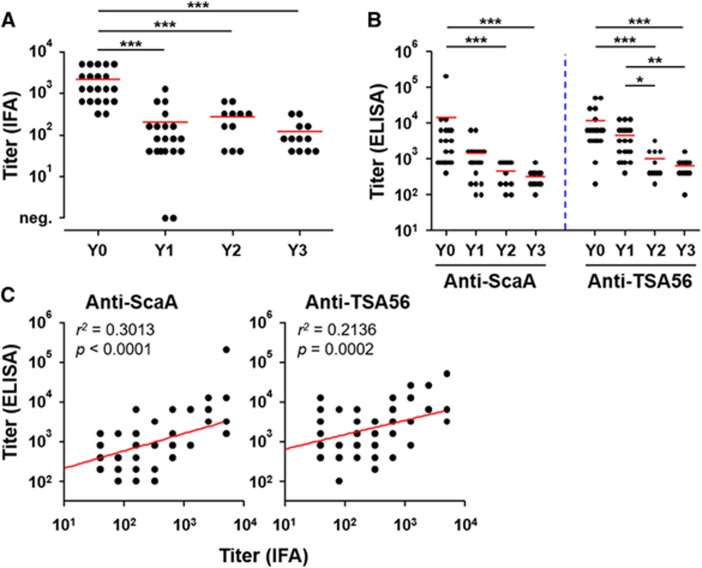
Kinetics and magnitude of antibody responses against bacterial antigens in scrub typhus patients. (**A**) IgG titers specific to whole bacterial antigens were measured by an immunofluorescence assay (IFA) using plasma samples from 64 scrub typhus patients. Patients were divided into four groups based on the time after recovery from the disease; Y0: within 2 weeks after recovery (*n*=21), Y1–Y3: 1–3 year(s) after recovery (*n*=20, 11 and 12, respectively). (**B**) Kinetic responses of IgG antibodies against ScaA or TSA56 were assessed by enzyme-linked immunosorbent assay (ELISA). The patient groups are the same as in **A**. The mean titer of the antibody responses (**A**, **B**) are indicated as red lines, and statistical analysis was performed using one-way analysis of variance (ANOVA) followed by the Newman–Keuls *t*-test for comparisons of the values among different groups (*P<0.05; **P<0.01; ***P<0.001). (**C**) Correlations of the IFA and ELISA titers were assessed by linear regression analysis (red lines), and the *r*^2^ and *P* values are shown in the graphs.

As measured by IFA, the mean titer of *O. tsutsugamushi*-specific IgG responses was 2194, ranging from 320 to 5120 (s.d.: ±1825), in 21 patients’ plasma collected within 2 weeks after recovery (Y0, Figure 1A). The antibody titers significantly dropped 1 year after recovery (Y1) and generally waned over 3 years (mean titer: Y1=204, Y2=272 and Y3=120). Specific antibody responses against the ScaA and TSA56 antigens, as measured by ELISA using the bacterial antigens, also gradually declined (Figure 1B). The antibody titers against both antigens were significantly lower at 2 years after infection. It is also notable that the anti-ScaA responses (mean titers: Y0=14 421; Y1=1460; Y2=473 and Y3=325) more rapidly declined than the anti-TSA56 titers (mean titers: Y0=12 011; Y1=4500; Y2=1000 and Y3=642), indicating that anti-TSA56 antibodies are more persistent than the anti-ScaA responses. Both antibody responses measured by ELISA were significantly correlated with the IFA titer against whole bacterial antigens, although the anti-ScaA titers showed better correlation with the IFA titer (Figure 1C).

### Differential kinetics and magnitude of specific IgG isotype responses against ScaA and TSA56

**Figure 2:**
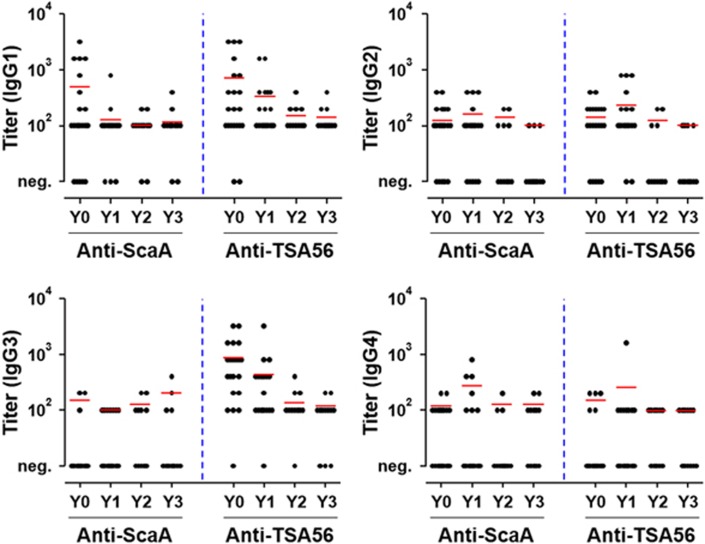
Kinetics and magnitude of IgG subclasses specific to ScaA and TSA56. The kinetic responses of IgG subclasses specific to ScaA or TSA56 in the plasma of recovered patients were measured by ELISA. The patient groups are the same as in Figure 1. The mean titer of the specific antibody responses are indicated as red lines. Y0 (*n*=21), Y1 (*n*=19), Y2 (*n*=11) and Y3 (*n*=12).

The distribution of IgG subclasses specific to ScaA and TSA56 was examined by ELISA. As seen in Figure 2, TSA56-specific IgG1 and IgG3 subclasses were dominant in Y0 patients, and the titers of both isotypes gradually declined thereafter. By contrast, IgG3 responses specific to ScaA antigen were barely detectable in the recovered patients, and only the IgG1 isotype was prevalent immediately after recovery (Y0). The ScaA-specific IgG1 responses also rapidly dropped at 1 year after recovery. IgG2 and IgG4 subclasses specific to the bacterial antigens were not significantly detected in recovered patients. Therefore, the dominant IgG isotype responses were IgG1 and IgG3 subclasses specific to TSA56, which declined gradually, as well as the IgG1 isotype against ScaA, which waned to basal levels within 1 year after infection.

### Kinetics and magnitude of antigen-specific T-cell responses

**Figure 3:**
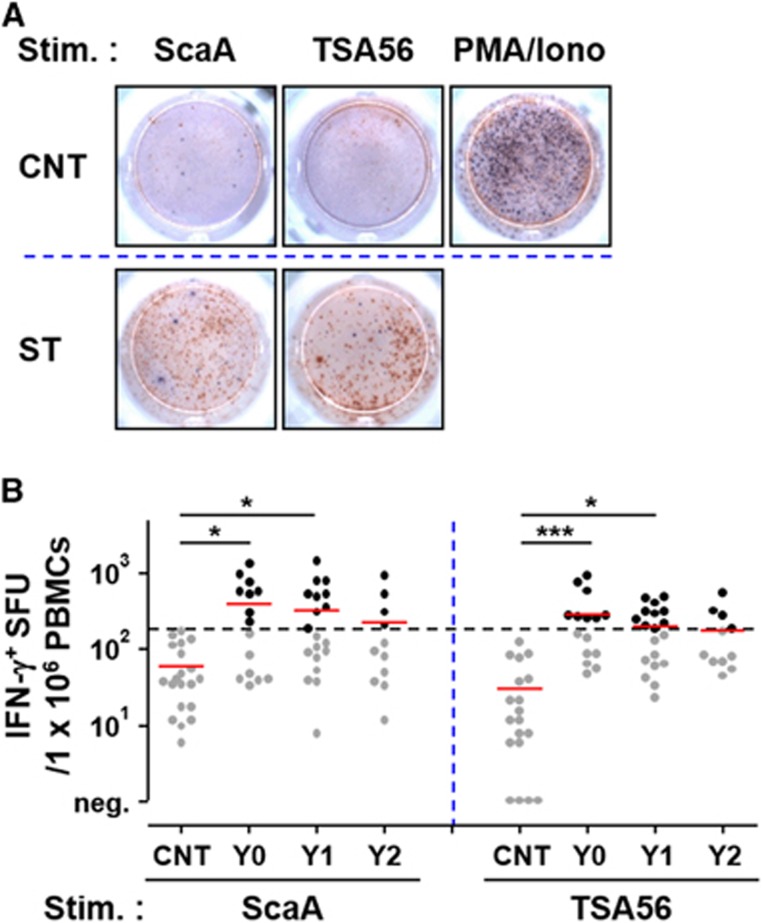
Kinetics and magnitude of antigen-dependent cellular responses. (**A**) Representative results of a human IFN-γ/IL-4 dual color ELISPOT assay are presented. Human peripheral blood mononuclear cells (PBMCs, 5 × 10^5^ cells per well) isolated from patients were stimulated with 10 μg/mL of the ScaA or TSA56 antigen in the presence of co-stimulatory anti-CD28 and anti-CD49d monoclonal antibodies at 37 °C for 18 h, and cellular spots were stained with anti-IFN-γ (brown) or IL-4 (blue) specific antibodies. Stim., stimulation; PMA/Iono, Phorbol myristate acetate (20 ng/mL)/ionomycin (1 μg/mL) as a positive control; CNT, PBMCs from the control group; ST, PBMCs from scrub typhus patients. (**B**) Kinetic responses of IFN-γ secretion by mononuclear cells after stimulation with the ScaA or TSA56 antigen were assessed by an ELISPOT assay as described in **A**. Cellular responses of PBMCs from 19 control individuals were used as a negative control (CNT), and the positive ELISPOT results (counts over the mean plus 3 × s.d. (dashed line) from 19 control samples) are indicated as black circles. The mean counts of the ELISPOT results are indicated as red lines, and statistical analysis was performed using ANOVA followed by the Newman–Keuls *t*-test for comparisons of the values among different groups (*P<0.05; ***P<0.001). Y0 (*n*=15), Y1 (*n*=18) and Y2 (*n*=11).

**Figure 4:**
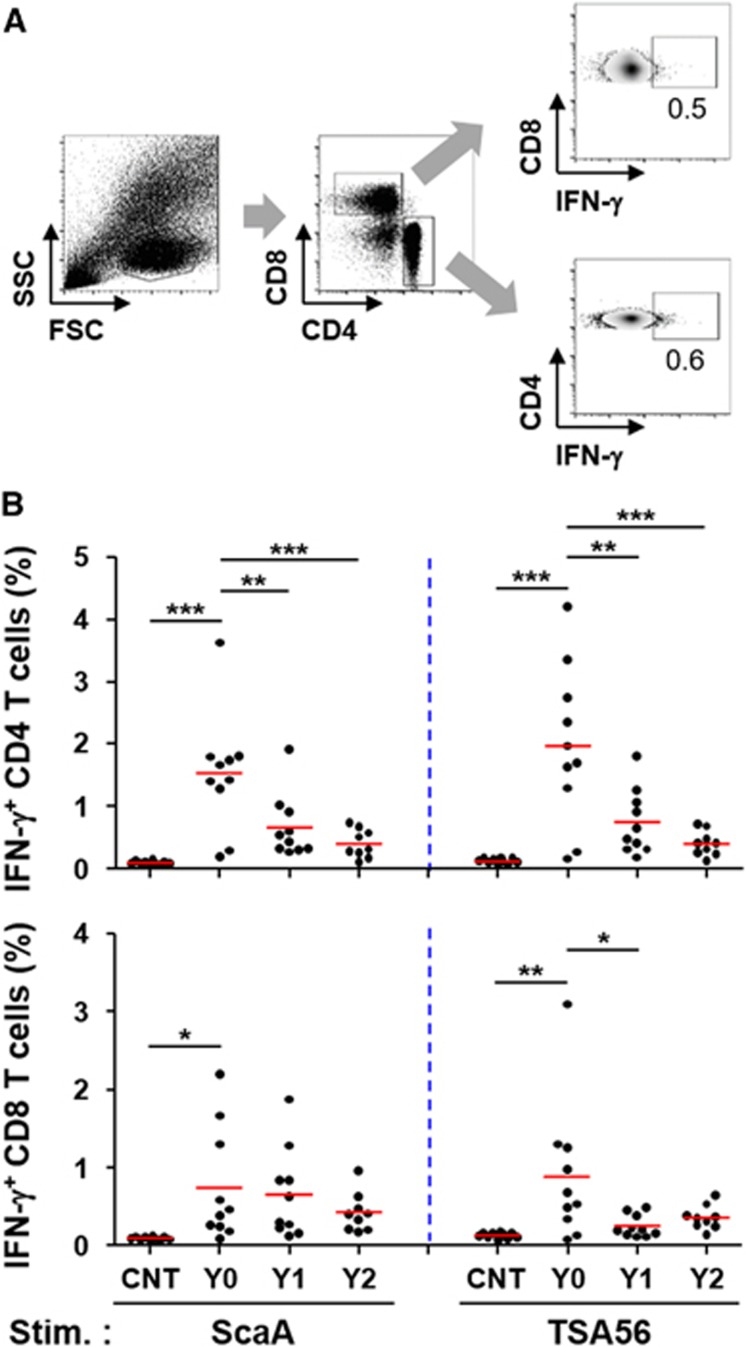
Kinetics and magnitude of ScaA- and TSA56-specific CD4 and CD8 T-cell responses. (**A**) Human PBMCs were isolated from control individuals and recovered patients and analyzed for antigen-specific T-cell responses by flow cytometry. Gating strategies for the analysis of CD4 or CD8 T cells secreting IFN-γ in an antigen-dependent manner are presented. (**B**) The kinetic changes of ScaA- or TSA56-specific T cells in the scrub typhus patients were measured by flow cytometry and compared with that of control individuals. The mean values of the relative percentile of IFN-γ-positive T cells among the CD4 or CD8 T-cell population are indicated as red lines, and statistical analysis was performed using ANOVA followed by the Newman–Keuls *t*-test for comparisons of the values among different groups (*P<0.05; **P<0.01; ***P<0.001). CNT (*n*=10), Y0 (*n*=10), Y1 (*n*=10) and Y2 (*n*=9).

To assess the relative magnitude and longevity of antigen-specific cellular immunity, we performed ELISPOT and flow cytometric analysis using PBMCs after stimulation with TSA56 and ScaA antigens. The cellular counts of IFN-γ-positive PBMCs stimulated with either of the antigens were significantly elevated in Y0 patients (mean counts: 196 for ScaA and 145 for TSA56/5 × 10^5^ PBMCs) and Y1 patients (mean counts: 167 for ScaA and 102 for TSA56/5 × 10^5^ PBMCs) compared to those of control patients (mean counts: 98 for ScaA and 71 for TSA56/5 × 10^5^ PBMCs) (Figure 3). At Y0, 53% (8 out of 15) and 67% (10 out of 15) of the recovered patients showed positive ELISPOT results (counts over the mean plus 3 × s.d. of 19 control samples) when stimulated with ScaA and TSA56, respectively. The counts of IFN-γ-positive PBMCs stimulated with either of the antigens gradually declined thereafter and were not statistically significant in the Y2 group. The cellular counts of IL-4-positive PBMCs upon antigen stimulation were not significantly different among the patient and control groups (Supplementary Figure S1), demonstrating that Th1-biased cellular responses are dominant in scrub typhus patients. To examine the antigen-specific T-cell response profile in more detail, CD4 and CD8 T cells secreting IFN-γ upon antigen stimulation were further assessed by flow cytometric analysis (Figure 4). Upon ScaA or TSA56 stimulation, cellular fractions of IFN-γ-positive CD4 T cells in PBMCs were significantly elevated in the Y0 group compared to those of the control, but were significantly reduced in recovered patients of the Y1 and Y2 groups. In the case of CD8 T cells, the IFN-γ-secreting population significantly increased upon antigen stimulation in the Y0 group. ScaA-specific, IFN-γ-positive CD8 T cells gradually declined without statistical significance in the Y1 and Y2 groups, whereas cells specific to TSA56 significantly decreased at 1 year after infection. These results indicate that antigen-specific T cells were significantly elevated immediately after recovery and gradually declined thereafter, as seen in the antibody responses. It is also notable that ScaA-specific CD8 T cells appear to be more persistent than those specific for TSA56.

### Screening of CD8 T-cell epitopes in the TSA56 antigen

TSA56, a major outer membrane protein of *O. tsutsugamushi*, has been a major target for vaccine development.^1^ In addition, it was recently reported that CD8 T cells provide immune protection against *O. tsutsugamushi* infection.^17, 18^ Therefore, assessment of *O. tsutsugamushi*-specific CD8 T-cell responses can be crucial for monitoring protective immunity in humans. To generate MHC class I-restricted epitopes in the TSA56 antigen as a tool for measuring CD8 T-cell responses, we screened potential HLA-A*0201-restricted CD8 T-cell epitopes in the TSA56 antigen using four web-based programs. Our analysis revealed 17 potential epitopes that were predicted with top 1% scores or percentile ranks in at least three of the four programs (Table 2). When we performed ELISPOT assays using the 17 peptide epitopes, two epitope peptides (YLRNISAEV and QLYKDLVKL) elicited significantly more IFN-γ-positive cellular spots in PBMCs from recovered patients with HLA-A2 type compared to control patients with HLA-A2 type and/or recovered patients with non-HLA-A2 type. Although our screen included a limited number of human subjects (3–5 per group) and needs to be further evaluated, the selected epitopes may be useful tools for monitoring antigen-specific CD8 T-cell immunity in recovered scrub typhus patients or for future vaccine study.

## DISCUSSION

In this study, we investigated the kinetics and magnitude of adaptive immunity, including antibodies and T-cell responses specific to the ScaA and TSA56 antigens in scrub typhus patients. These two outer membrane proteins have been shown to have a significant role in bacterial adhesion and invasion.^16, 27, 28^ In addition, immunization with the two antigens could provide protective immunity, potentially via generation of neutralizing antibodies and cellular immunity.^15, 16, 29, 30^ As the nature of protective immunity against the intracellular pathogen has been primarily assessed in murine infection models, systemic characterization of the antibody and T-cell responses that are specific to the bacterial antigens in humans is urgently required for the development of an effective scrub typhus vaccine.

Antibody responses to ScaA and TSA56 in recovered scrub typhus patients were generally short-lived and waned to baseline levels 2 years after infection (Figures 1 and 2). The levels and trends of the adaptive immune responses were generally consistent regardless of patients’ age or gender (data not shown). This short longevity of IgG responses against the TSA56 antigen has also been reported in other studies.^22, 31^ Nevertheless, specific antibodies against TSA56 were more robust and persistent than anti-ScaA antibodies. The relative robustness of the IgG responses against TSA56 compared to other autotransporter proteins, including ScaA, was also previously confirmed in scrub typhus patients.^23^ These results might be because TSA56 is the most abundant major outer membrane protein, occupying ~20% of the whole bacterial proteome.^32^ However, the relative immunogenicity of the two membrane antigens needs to be further characterized. It is also interesting to note that anti-the TSA56 IgG responses were predominantly composed of IgG1 and IgG3 subclasses, whereas IgG responses specific to ScaA primarily consisted of transient IgG1 and limited responses by the other subclasses (Figure 2). Considering that IgG1 and IgG3, particularly IgG3 due to its longer hinge region and greater flexibility, are more potent triggers of effector mechanisms such as neutralization, complement fixation and phagocytosis than IgG2 and IgG4,^19^ stronger and more persistent induction of IgG1 and IgG3 against both of the membrane antigens might be required for better protection against *O. tsutsugamushi* infection. However, the role of antibody responses in protective immunity is still unclear in animal infection models. Passive transfer of convalescent sera did not protect the majority of mice challenged with *O. tsutsugamushi*, but pre-incubation of the bacterial pathogen with immune sera markedly increased the survival rate of infected mice.^33^ In addition, the presence of specific antibodies did not terminate rickettsemia in a non-human primate infection model.^34^ *O. tsutsugamushi* and specific antibodies circulated concurrently in infected primates, suggesting that a mechanism other than humoral immunity might be an important factor in the eradication of the pathogen *in vivo*.^34^ Recently, two reports showed that genetic knockout or antibody-mediated depletion of CD8 T cells increased the bacterial loads and severity of illness in murine infection models.^17, 18^ Adoptive transfer also confirmed the major role of immune CD8 T cells in protecting against *O. tsutsugamushi* infection. Although the relative contribution of CD4 T cells or antibody-secreting B cells has not been directly compared with CD8 T-cell activity, the critical importance of CD8 T cells in effective host immune responses during bacterial infection is convincing in murine infection models. Previously, we and others showed a preferential increase of CD8 T cells with activated phenotypes in scrub typhus patients during the convalescent phase,^6, 35^ suggesting a potential role of cytotoxic T cells in clearing bacteria during human infection. However, the antigen-specificity of induced T cells has never been assessed in human infections. In this study, we demonstrate for the first time a significant elevation of ScaA- and TSA56-specific CD4 and CD8 T cells secreting IFN-γ in scrub typhus patients within 2 weeks after recovery (Figure 4). Consistent with this result, dominance of Th1-biased immune responses, as represented by elevated IFN-γ (Figure 3), has also been observed in scrub typhus patients.^36, 37^ In summary, cellular immunity mediated by antigen-specific T cells may have a significant role in the eradication of bacterial pathogens from infected patients. ScaA- or TSA56-specific T cells, however, rapidly declined at 1 year after infection and were barely detectable at 2 years after recovery. This short longevity of antigen-specific T cells, as well as antibody responses, might be clinically associated with the limited memory responses observed in recovered or vaccinated patients.^38, 39, 40, 41^

**Figure 5:**
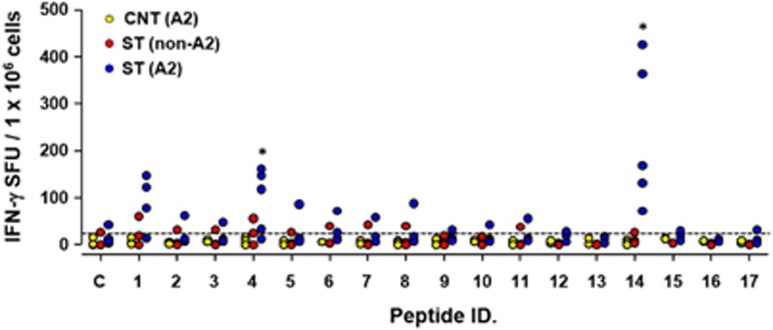
Screening and selection of HLA-A*0201-restricted CD8 T-cell epitopes for the TSA56 antigen. Human peripheral blood mononuclear cells (PBMCs, 5 × 10^5^ cells per well) were isolated from control individuals with HLA-A2 type (CNT, yellow dots, *n*=3), recovered patients with non-HLA-A2 type (ST (non-A2), red dots, *n*=3), or patients with HLA-A2 type (ST (A2), blue dots, *n*=5) and then stimulated with 17 different epitope peptides (1 μM, amino acid sequences summarized Table 2) in the presence of co-stimulatory anti-CD28 and anti-CD49d monoclonal antibodies at 37 °C for 18 h. Cellular spots were stained with anti-IFN-γ specific antibodies and counted. Positive cellular counts were significantly higher in PBMCs stimulated with peptide #4 and #14 among the 17 predicted peptides (*P<0.05). The number of samples for each group varied (2–5 samples per group) due to the limited availability of PBMCs.

Finally, we also screened and selected epitope peptides for the analysis of TSA56-specific CD8 T cells. Potential HLA-A*0201-restricted CD8 T-cell epitopes on the TSA56 antigen were first screened bioinformatically as TSA56 is one of the most common HLA alleles in the human population, especially in the endemic region of scrub typhus (http://www.allelefrequencies.net/). Two epitope peptides (YLRNISAEV and QLYKDLVKL) elicited specific and a significantly higher number of IFN-γ-positive cellular spots in PBMCs from recovered patients with HLA-A2 type, as measured by the ELISPOT assay (Figure 5). These peptide epitopes may therefore be useful for the analysis of TSA56-specific cellular immunity in humans with HLA-A2 type. Interestingly, the predicted T-cell epitopes are primarily located outside of the variable domains of TSA56 (Supplementary Figure S2).^32^ By contrast, antigenic determinants for antibody responses generally overlap with variable domains of TSA56,^42, 43^ and immunological pressure on the major outer membrane protein during vertebrate host infection might be a crucial driver of genetic diversification of *O. tsutsugamushi*.^1^ Discovery of the preservation of T-cell epitopes in the major outer membrane protein among different genotypes represents a significant advancement toward generating universal and protective T-cell responses that encompass the diverse genotype strains of *O. tsutsugamushi*. The potential role of T-cell responses specific to the conserved epitopes in protective immunity against the bacterial infection needs to be further verified for the development of a universal scrub typhus vaccine.

## Figures and Tables

**Table 1:** List of primers used in this study

**Gene**	**Primer sequences**^a^	**Product size (bp) (amplified region in nt position)**^b^
*scaA*	Forward: CG*GGATCC*GATCCATCAGCTTCATCA	2913 (88–3000)
	Reverse: CG*GTCGAC*TATATCTTCGTCTTTGCC	
*tsa56*	Forward: CG*GAATCC*GCACCAGGATTTAGAGCA	981 (250–1230)
	Reverse: CG*GTCGAC*TTTACCTTGATTCTTTGC	

a Restriction enzyme sites are italisized.

b *O. tsutsugamushi* Boryong strain served as the template.^44^

**Table 2:** Predicted HLA-A*0201-restricted CD8+ T-cell epitopes of TSA56

**Peptide ID**	**Start position in TSA56**^a^	**Peptide sequence**	**M.W.**	**IC**_**50**_ **(nM)**^b^
1	5	MLIASAMSA	893.11	42.25
2	6	LIASAMSAL	875.07	508.87
3	93	VMYLRNISA	1065.28	260.18
4	95	YLRNISAEV	1063.19	21.60
5	133	KLTPPQPTM	1011.22	1249.17
6	145	SIADRDFGI	992.07	110.68
7	201	HMMVNPVLL	1052.35	87.23
8	203	MVNPVLLNI	1011.27	95.12
9	242	SLVVGLAAL	841.03	110.68
10	263	VLSDKIIQI	1027.24	13.28
11	303	KIQELGDTL	1015.14	1785.20
12	306	ELGDTLEEL	1017.07	1376.93
13	366	RLLNGSDQI	1014.12	109.48
14	376	QLYKDLVKL	1118.35	124.66
15	436	SMVVGQVKL	959.19	631.81
16	443	KLYADLVTT	1022.18	42.25
17	482	MVASGALGV	802.96	91.09

a Amino acid position in TSA56 sequence (GeneBank accession No. CAM79668).

b Peptide affinity predicted by NetMHCpan 3.0 Server.^26^
