# Supplementary material for: Longevity of antibody and T-cell responses against outer membrane antigens of Orientia tsutsugamushi in scrub typhus patients
Source: Emerg Microbes Infect. 2017 Dec 20;6(12):e116–. doi: 10.1038/emi.2017.106 (PMC5750460; doi:10.1038/emi.2017.106)

**Supplementary Figure S1** Kinetics and magnitude of antigen-dependent cellular responses secreting IL-4. Human peripheral blood mononuclear cells (PBMCs, 5 × 10^5^ cells/well) isolated from control individuals and the patients were stimulated with 10 μg/ml of ScaA or TSA56 antigen in the presence of co-stimulatory anti-CD28 and anti-CD49d monoclonal antibodies at 37°C for 18 h and cellular spots were counted as described in Figure 3. Stim.: stimulation, CNT: PBMCs from control group, CNT (*n* = 19), Y0 (*n* = 15), Y1 (*n* = 18), and Y2 (*n* = 11).


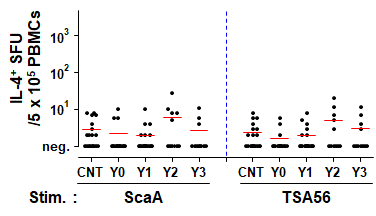

Supplement: Supplementary Figure 1 [file emi2017106x1.doc]
