# Supplementary material for: Longevity of antibody and T-cell responses against outer membrane antigens of Orientia tsutsugamushi in scrub typhus patients
Source: Emerg Microbes Infect. 2017 Dec 20;6(12):e116–. doi: 10.1038/emi.2017.106 (PMC5750460; doi:10.1038/emi.2017.106)
Supplement: Supplementary Information [file emi2017106x2.doc]

**Supplementary Figure S2** Amino acid sequences and position of the 17 predicted epitope peptides (#1 ~ #17, red squares, summarized in Table 2) in nine aligned TSA56 sequences from the indicated genotypes (Boryong, Gilliam, Ikeda, Karp, Kato, Kawasaki, Saitama, Shimokoshi, and TA763). Blue squares indicate the four variable domains (VD I ~ IV) as defined in a previous study^1^ and NCBI accession no. of each genotype sequence is indicated with brackets.


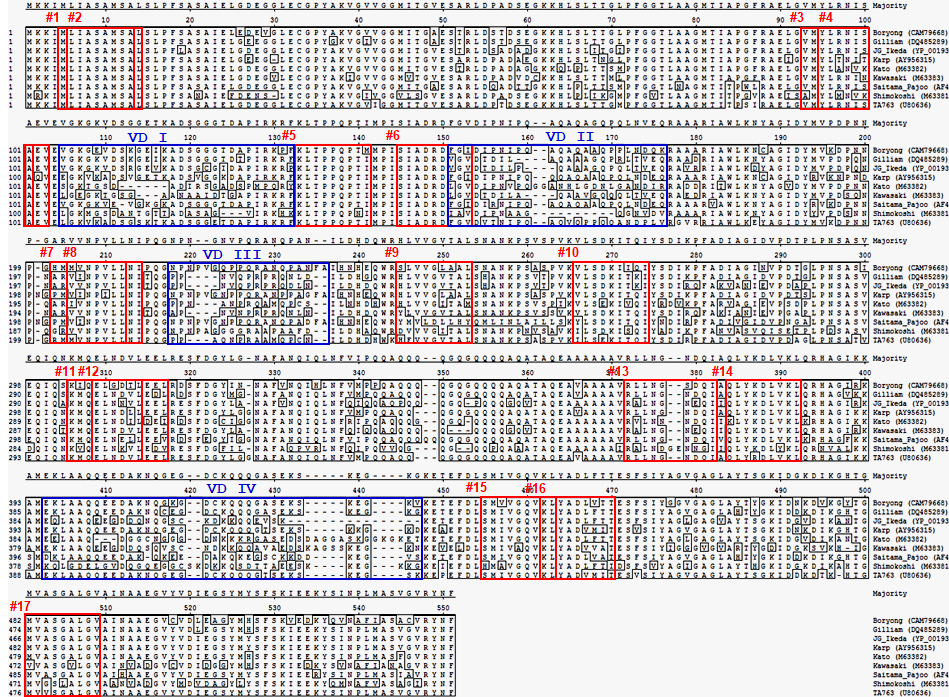


**Reference.**

1. Ohashi N, Nashimoto H, Ikeda H, Tamura A. Diversity of immunodominant 56-kDa type-specific antigen (TSA) of Rickettsia tsutsugamushi. Sequence and comparative analyses of the genes encoding TSA homologues from four antigenic variants*. J Biol Ch*em 1992 Jun 25**; 2**67(1**8**): 12728-12735.
